# Return of Sexual Activity Within Six Weeks of Childbirth Among Married Women Attending Postpartum Clinic of a Teaching Hospital in Ethiopia

**DOI:** 10.3389/fmed.2022.865872

**Published:** 2022-04-25

**Authors:** Dejene Edosa Dirirsa, Mukemil Awol Salo, Tariku Regea Eticha, Tinsae Abeya Geleta, Berhanu Senbeta Deriba

**Affiliations:** ^1^Department of Midwifery, College of Health and Medical Sciences, Salale University, Fitche, Ethiopia; ^2^Department of Public Health, College of Health and Medical Sciences, Salale University, Fitche, Ethiopia

**Keywords:** postpartum sexual activity, Ethiopia 2022, sexual resumption, early sexual activity, return to sex, sexual activity, six weeks of childbirth, Ethiopia

## Abstract

**Introduction:**

Postpartum sexual health gets very little attention compared to pregnancy and childbirth, even though most maternal deaths and disabilities occur during this time. Therefore, the study aimed to assess return of sexual activity within 6 weeks of childbirth among married women attending postpartum clinic of a teaching hospital in Ethiopia, 2021.

**Methods:**

The hospital-based cross-sectional study design was implemented from September to October 2021. Eligible postpartum women were sampled by systematic random sampling technique. The data was entered into EPI-info and exported to SPSS version 24 for further analysis. All variables with a *p*-value < 0.05 in multivariable analyses were taken as associated factors with the return to sexual activity before 6 weeks of childbirth.

**Results:**

A total of 421 postpartum women participated in the study. The prevalence of women who return to sexual activity 6 weeks after childbirth was 31.6%. The study revealed that monogamy (Adjusted Odds Ratio (AOR) = 4.4, 95% Confidence Interval (CI) (2.1, 9.4)), parity (AOR= 0.11, 95% CI (0.02–0.81)) and (AOR = 0.1, 95% CI (0.015–0.72)), postnatal care (AOR= 1.8, 95% CI (1.01–3)) and infants feeding status (AOR=2.3, 95% CI (1.3–4)) were significantly associated with return of sexual activity before 6 weeks of childbirth.

**Conclusion:**

The findings of this study suggested that, nearly one-third of postpartum women engaged to sexual activity within 6 weeks of childbirth. Return of sexual activity within 6 weeks of childbirth associated with monogamy type of marriage, parity, postnatal care, and child feeding status. Strengthening postpartum counseling regarding the appropriate time to resume sexual activity is crucial. Regular postpartum visits following deliveries should also be encouraged by health care providers.

## Introduction

The postpartum period has received less attention than pregnancy and childbirth, despite the fact that it is the time when most maternal deaths and disabilities occur (1). As sexual health undergoes fundamental changes during pregnancy, childbirth, and the postpartum period, postpartum sexual health is one of the emerging international issues. The World Health Organization (WHO) recommends that one of the most important issues to be addressed during the postpartum period is postpartum sexual health (2). Arousal, libido, orgasm and sexual pleasure are all signs of good sexual health after childbirth (3).

In fact there is a positive relationship between sexual and marital satisfaction which develops true relationships and benefits both partners in strengthening the family (4). However, pregnancy and the time after childbirth have a negative impact on women's and their partner's sexuality (5). The interest in sexual activity decreases during pregnancy but returns to normal soon after an average of 6 weeks after childbirth (6). It has lately been suggested that it is safe to resume postpartum sexual activity at least 6 weeks after childbirth (7).

The postpartum sexual resumption after childbirth may reduce the likelihood of compromising family's relationships and reduce the family's' health at risk due to sexually transmitted infections such as HIV/AIDS through being polygamous (8). However, according to the World Health WHO and other studies, more than half (50%), of women resume sexual activity within 6 weeks of childbirth (9–11). For instance, in Turkey 79.1% and China 68.7% of postpartum women engage sexual activity within the first 6 weeks of childbirth (12, 13).

Early sexual resumption is also common in African countries with varying and/or unpredictable durations such as Nigeria 38.7% and Uganda 21.6% (14–17). Studies in Ethiopia revealed that many women resumed sexual activity within 6 weeks of childbirth: Nekemte (20.2%) (18), Gondar town (26.9%) (15, 19), Jimma and Addis Ababa city, more than half (>50%) of women started sexual intercourse earlier after childbirth (3, 11).

It is generally recognized that after giving birth, women experience a variety of sexual health problems with variety of negative consequences such as wound infection, painful intercourse, vaginal dryness, inability to reach orgasm, vaginal laxity and decreased libido (3, 20, 21). While a temporary decrease in libido following childbirth is to be expected, women should not feel pain during sexual activity. If these symptoms are not addressed, there will be a fear of having sexual relations, and the problem will increase, resulting in physical and psychological illness (22).

In addition, early postpartum sexual resumption without using contraception can result in an unexpected pregnancy. This may result in poor maternal and infant health, as well as a high risk of death for infants under the age of 1 year (23). Previous studies showed that income, type of marriage, sexual intercourse during pregnancy, mode of delivery, contraceptive method utilization, parity, educational status, desire for additional baby, demand of the partner for intercourse and residence are factors associated with the early return to postpartum sexual activity (3, 7, 11, 13, 18).

In Ethiopia; sexual health after childbirth like when it is safe to resume sexual intercourse is not properly addressed in which health care practitioners rarely talk to postpartum women about sexual issues (3, 18). The majority of Ethiopian studies focused on the use of contraception to avoid rapid repeat pregnancies in order to resolve women's health problems following childbirth. This indicates the gaps of information on the factors associated with early postpartum sexual resumption after childbirth (18). Therefore, this study aimed to assess return of sexual activity within 6 weeks of childbirth among married women attending postpartum clinic of Salale University Comprehensive Specialized Teaching Hospital in Ethiopia, 2021.

## Materials and Methods

### Study Design, Period and Area

The hospital based cross-sectional study design was employed from September to October 2021. The study was conducted at Salale University Comprehensive Specialized Teaching Hospital. The hospital is located in Fiche Town, North Shewa District, and 112 kilometers away from Addis Ababa. It is one of the first-line hospitals in the North Shewa district, serving more than 1.5 million people each year and providing more than 3,978 postnatal services (PNC) per year.

### Population

The source population of this study was postpartum women who went to Salale University Comprehensive Specialized Teaching Hospital for postpartum and child immunization services. The study population was postpartum women attending postpartum and child immunization services at Salale University Comprehensive Specialized Teaching Hospital during study period. All married postpartum women who visited postpartum or child immunization clinic during data collection period were included in the study. Women who were critically ill during the study period were excluded from the study.

### Sample Size Determination and Sampling Procedure

The single population proportion formula used to calculate the sample size, with a 95% confidence level and 5% margin of error, the proportion (p) of early postpartum sexual intercourse 53.9% taken from a similar study conducted in Jimma district / Ethiopia (3). Finally, 421 eligible postpartum women were sampled by systematic random sampling technique. Individual clients who visited a postnatal clinic were approached using a sampling interval of K = 10 [N/n, where N is the average number of clients who visited a postnatal clinic and n is the computed final sample size]. The first participant was chosen using a lottery method based on the obtained interval.

### Data Collection Instruments and Techniques

A semi-structured questionnaire was developed after a thorough review of the literature. The questionnaire was initially written in English, and then translated into Afan Oromo and again retranslated back to English to check for any inconsistencies or distortions in the meaning of terms and concepts. Socio-demographic characteristics, reproductive and maternal health service use-related characteristics, post-natal care-related factors, and sexual health during pregnancy and after birth-related characteristics were all included in the questionnaire. Data were collected using a semi-structured questionnaire in the Afan Oromo language version after pre-testing on 5% of the same-origin population outside the sample population. The pre-testing process was carried out in Muke Turi primary hospital, which accounted for 5% of the total sample size. The tool reliability test was checked by Cronbach's alpha, which indicated that 0.87. Two midwives collected the data after being briefed on the research and data collection protocols.

### Data Processing and Analysis

The data entered into EPI-info and then exported to SPSS version 24 for further analysis. Descriptive analysis (such as frequencies, percentages, means, and standard deviation) and inferential analysis was performed. Bivariate and multivariable logistic regressions used to check the significance of the outcome variable and each of the independent variable using odds ratio with 95% confidence interval. All variables with *p*-value < 0.25 at bivariate was considered as a candidate for multivariable analysis and those variables with a *p*-value < 0.05 in multivariable analyses were taken as statistical significant associated factors with return to sexual activity before 6 weeks of childbirth. Multivariable logistic regression analyses were performed to control for possible confounding effects of the selected variables. The main assumption of the binary logistic regression model was tested. The assumption result showed that there is no existence of a significant effect modification. The multicollinearity among the independent variables was also evaluated using a multiple linear regression model. The evaluation result does not indicate evidence of multicollinearity. Model goodness-of-fit was tested by the Hosmer-Lemeshow model and the forward stepwise (likelihood ratio) method used. The *p*-value for the model fitness test was 0.935.

### Variables

Dependent variable: return to sexual intercourse before 6 weeks of childbirth.

Independent variables: socio demographic characteristics (polygamy, educational status, occupation, age), reproductive and maternal health service use-related characteristics (gravidity, index of current pregnancy status, mode of delivery, place birth), post natal care related factors (postnatal care visit, current infant feeding status, resumption of menstruation), sexual health during pregnancy and after birth related characteristics (Practice of sexual intercourse during pregnancy, Last sex trimester, Reason to resume sexual intercourse).

### Operational Definitions

#### The Time of Return to Sexual Activity

In this study the category given for return to sexual activity before 6 weeks of childbirth as code 1 and return to sexual activity after 6 weeks of childbirth as code 0.

#### Index of Current Pregnancy Status

Intention to have the child before conception.

#### Resumption of Menses After Delivery

Is the first menstrual cycle after childbirth.

### Ethics Approval and Informed Consent

The research was carried out in agreement with the Helsinki Declaration. Salale University Ethical Review Committee authorized the ethical clearance of written consent for those over the age of 18 and an agreement form for those under the age of 18 from their partners with Ref. No. SLUERC/122/2021. Official authorization was received from the selected hospital, as well as a support letter from the North Showa Zone health office. After describing the study's goal to participants, written agreement was obtained. The privacy and anonymity of study subjects were preserved by omitting their names from the questionnaire and storing their data in a secure location.

**Figure 1:**
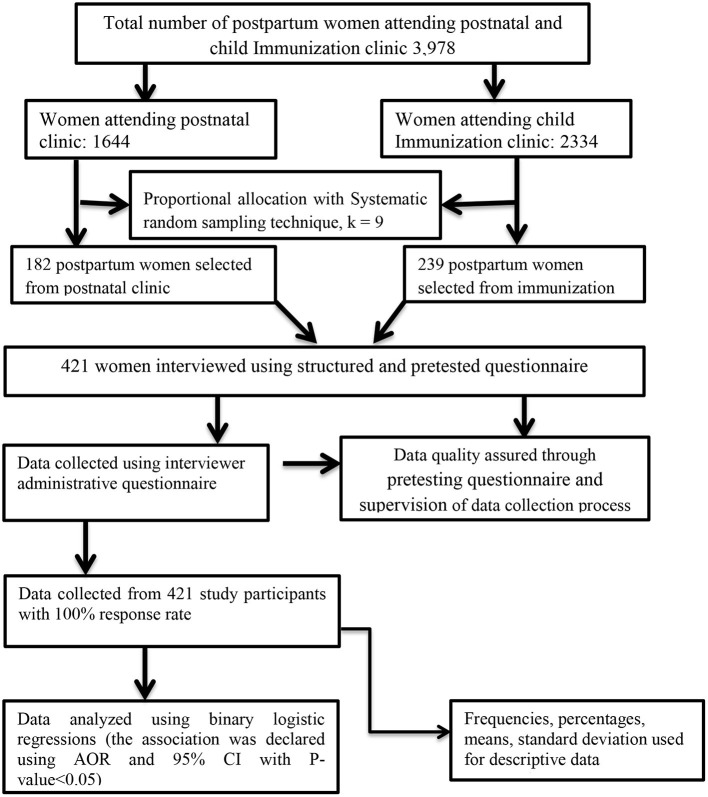
Flow chart of materials and methods.

The following figure illustrates the major methods and materials section (Figure 1).

## Results

### Socio-Demographic Characteristics Among Married Post-partum Women at Salale University Comprehensive Specialized Teaching Hospital, From September to October 2021 (*n* = 421)

A total of 421 postpartum women participated in the study. The mean age of participants was 28.54 years (SD ± 5.08), and a majority of participants, 318 (75.5%), were of Oromo ethnicity. About 292 (69.4%) of the participants were monogamous, and nearly half, 213 (50.6%) of the participants had been living with their husbands for only 1 to 2 years. The majority, 297 (70.5%) of study participants lived in cities, accounting for more than two-third of women participated in the study, and 40.4% of postpartum women had a college education or higher (*See* Table 1).

**Table 1:** Socio-demographic characteristics of post-partum women at Salale University comprehensive specialized teaching hospital, September to October 2021 (*n* = 421).

**Variables**		**Return to sexual intercourse after childbirth**		**Total**
		** <6 weeks**	**>6 weeks**	
Maternal age (in years)	15–19	6 (1.4%)	0	6 (1.4%)
	20–24	29 (6.9)	59 (14%)	88 (20.9%)
	25–29	41 (9.7%)	111 (26.4%)	152 (36.1%)
	30–34	33 (7.8%)	78 (18.5%)	111 (26.4%)
	35–49	24 (5.7%)	40 (9.5%)	64 (15.2%)
Ethnicity	Oromo	95 (22.6%)	223 (53%)	318 (75.5%)
	Amhara	34 (8.1%)	60 (14.3%)	94 (22.3%)
	Tigre	2 (0.5%)	0	2 (0.5%)
	Other ^*^	2 (0.5%)	5 (1.2%)	7 (1.7%)
Religion	Muslim	12 (2.9%)	28 (6.6%)	40 (9.5%)
	Orthodox	103 (31.9%)	220 (52.3%)	323 (76.7%)
	Protestant	15 (3.6%)	38 (9%)	53 (12.6%)
	Catholic	3 (0.7%)	2 (0.5%)	5 (1.2%)
Polygamy	Yes	20 (4.7%)	109 (25.9%)	129 (30.6%)
	No	113 (26.9%)	179 (42.5%)	292 (69.4%)
Maternal educational status	No formal education	22 (5.2%)	36 (8.6%)	58 (13.8%)
	Primary	34 (8.1%)	70 (16.6%)	104 (24.7%)
	Secondary	23 (5.5%)	66 (15.7%)	89 (21.1%)
	College and above	54 (12.8%)	116 (27.6%)	170 (40.4%)
Maternal occupation	Government employee	41 (9.7%)	89 (21.1%)	130 (30.9%)
	Self-employee	30 (7.1%)	59 (14%)	89 (21.1%)
	Housewife	52 (12.4%)	126 (29.9%)	178 (42.3%)
	Others ^**^	10 (2.4%)	14 (3.3%)	24 (5.7%)
Residence	Urban	94 (22.3%)	203 (48.2%)	297 (70.5%)
	Rural	39 (9.3%)	85 (20.2%)	124 (29.5%)
Duration of living together with husband	1–2 years	74 (17.6%)	139 (33.0%)	213 (50.6%)
	3–5 years	35 (8.3%)	77 (18.3%)	112 (26.6%)
	>5 years	24 (5.7%)	72 (17.1%)	96 (22.8%)

Others ^*^ = wolaita,

Gurage ^**^ = student, Non-governmental organization (NGO) employee and daily laborer.

### Reproductive and Maternal Health Service Related Characteristics Among Married Post-partum Women at Salale University Comprehensive Specialized and Teaching Hospital, From September to October 2021 (*n* = 421)

According to the result of this study, more than three-quarters of the participants, 366 (86.9%), gave birth to their first child during the current delivery. Almost all, 399 (94.8%) of the study participants' index of the pregnancy status was planned and about 405(96.2%) had prenatal care during their last pregnancy. The majority, 292 (69.4%) of women gave birth their last baby in a hospital. Regarding women's mode of delivery in this finding, about three-quarters, 318(75.5%) give birth by spontaneous vaginal delivery (SVD) without an episiotomy during their last delivery and 264(62.9%) of them were multiparous (*See* Table 2).

**Table 2:** Reproductive and maternal health service related characteristics among married post-partum women at Salale University Comprehensive Specialized and teaching hospital, from September to October 2021 (*n* = 421).

**Variables**		**Return to sexual intercourse after childbirth**		**Total**
		**<6 weeks**	**>6 weeks**	
Number of pregnancies	Primi-gravid	120 (28.5%)	246 (58.4%)	366 (86.9%)
	Multi gravid	13 (3.1%)	42 (10.0%)	55 (13.1%)
History of abortion	Yes	17 (4.0%)	35 (8.3%)	52 (12.4%)
	No	116 (27.6%)	253 (60.1%)	369 (87.6%)
Index of current pregnancy status	Planned	129 (30.6%)	270 (64.1%)	399 (94.8%)
	Un planned	4 (1.0%)	18 (4.3%)	22 (5.2%)
ANC visit during your last pregnancy	Yes	128 (30.4%)	277 (65.8%)	405 (96.2%)
	No	5 (1.2%)	11 (2.6%)	16 (3.8%)
Obstetric related complication	Yes	8 (1.9%)	24 (5.7%)	32 (7.6%)
	No	125 (29.7%)	264 (62.7%)	389 (92.4%)
Place birth for the last delivery	Health center	41 (9.7%)	67 (15.9%)	108 (25.7%)
	Hospital	87 (20.7%)	205 (48.7%)	292 (69.4%)
	Private clinic	0 (0.0%)	6 (1.4%)	6 (1.4%)
	Home	5 (1.2%)	10 (2.4%)	15 (3.6%)
Mode of delivery	SVD without episiotomy	107 (25.4%)	211 (50.1%)	318 (75.5%)
	SVD with episiotomy	16 (3.8%)	45 (10.7%)	61 (14.5%)
	Caesarian Section	10 (2.4%)	32 (7.6%)	42 (10.0%)
Parity	Primi-Para	46 (11%)	102 (24.3%)	148 (35.2%)
	Multi-Para	82 (19.5%)	182 (43.3%)	264 (62.9%)
	Grand- Multi-Para	5 (1.2%)	3 (0.7%)	8 (1.9%)

### Post Natal Care Related Characteristics Among Married Post-partum Women at Salale University Comprehensive Specialized and Teaching Hospital, From September to October 2021 (*n* = 421)

The majority of women who participated in the study, 257 (61%) had postpartum care at their last delivery, and most of them, 279 (66.3%) had exclusively breastfed their infants. More than half, 233 (55.3%) of the study participants' menstruation cycle returned after a recent delivery, and 378 (89.8%) had a good understanding of any modern contraceptive method **(***See* Table 3).

**Table 3:** Post natal care related characteristics among married post-partum women at Salale University Comprehensive Specialized and Teaching hospital, from September to October 2021 (*n* = 421).

		**Return to sexual intercourse**	
**Variables**		**before 6 weeks of childbirth**		**Total**	**Chi-square**
		**<6 weeks**	**>6 weeks**		
Postnatal care visit for the last births	Yes	93 (22.1%)	164 (39.0%)	257 (61.0%)	0.011^*^
	No	40 (9.5%)	124 (29.5%)	164 (39.0%)	
Current infant feeding status	Exclusive breast feeding	98 (23.3%)	181 (43.0%)	279 (66.3%)	0.029^*^
	Formula Feeding	35 (8.3%)	107 (25.4%)	142 (33.7%)	
Resumption of menstruation after recent birth	Yes	77 (18.3%)	156 (37.1%)	233 (55.3%)	0.474
	No	56(13.3%)	132(31.4%)	188(44.7%)	
Knowledge about modern contraceptive	Yes	121 (28.7%)	257 (61.0%)	378 (89.8%)	0.583
methods	No	12 (2.9%)	31 (7.4%)	43 (10.2%)	

* * = Statistically significant at p <0.05*.

### Sexual Health Characteristics of Early Return to Postpartum Sexual Intercourse Among Married Post-partum Women at Salale University Specialized and Teaching Hospital, From September to October 2021 (*n* = 421)

The majority, 353 (83.8%) of the women practiced sexual intercourse during their last pregnancy and from those who practiced sexual intercourse during pregnancy most of them, 220 (62%) end up the sexual activity during the third trimester pregnancy.

Most of the women, 221 (52.5%) discussed about return to postpartum sexual activity with their husbands to initiate the sexual intercourse after recent childbirth, and 195 (46.3%) women resumed their sexual practices under the influence of their husbands.

According to the finding of this study, most of the women, 337 (80%) were not getting health education from health care providers regarding the time when to resume sexual intercourse after childbirth. Prevalence of women who return to sexual activity before 6 weeks after childbirth (non-recommended time) was 133 (31.6%) (*See* Table 4).

**Table 4:** Sexual health characteristics of study participants who attending Salale University specialized and teaching hospital, from September to October 2021 (*n* = 421).

		**Return to sexual intercourse**	
**Variables**		**before 6 weeks of childbirth**		**Total**	**Chi-square**
		**<6 weeks**	**>6 weeks**		
Practice of sexual intercourse during pregnancy	Yes	117 (27.8%)	238 (56.8%)	355 (84.3%)	0.202
	No	16 (3.8%)	50 (11.9%)	66 (15.7%)	
Last sex trimester	First trimester	11 (3.1%)	10 (2.8%)	21 (5.9%)	0.77
	Second trimester	41 (11.5%)	73 (20.6%)	114 (32.1%)	
	Third trimester	65 (18.3%)	155 (43.7%)	220 (62.0%)	
Reason to return to sexual activity	Husband demand for sex	64 (15.4%)	131 (31.1%)	195 (46.3%)	0.456
	Jointly discussion	67 (15.9%)	154 (36.6%)	221 (52.5%)	
	Others^*^	2 (0.5%)	3 (0.7%)	5 (1.2%)	
Help from health care provider	Yes	38 (9.0%)	67 (15.9%)	105 (24.9%)	0.242
	No	95 (22.6%)	221 (52.5%)	316 (75.1%)	
health education from health care provider after delivery	Get	33 (7.8%)	51 (12.1%)	84 (20.0%)	0.090
	Not get	100 (23.8%)	237 (56.7%)	337 (80.0%)	

Others^*^ = women's wish to have another child within a short period of time.

### Factors Associated With Return to Sexual Intercourse Before 6 Weeks of Childbirth Among Married Post-partum Women at Salale University Specialized Teaching Hospital, From September to October 2021 (*n* = 421)

In the bivariate logistic regression those variables with *p*-value of ≤ 0.25 considered as the candidate for multivariable logistic regression analysis, such as parity, index of current pregnancy status, mode of delivery, postnatal care visit for the last births, infant feeding status, last trimester sexual activity during pregnancy, getting help and health education from healthcare providers after giving birth.

In multivariable logistic regression monogamy, parity (multi-parous, grand- multi parous), postnatal care visit for the recent birth and infant feeding status were found to be significantly associated with return to sexual activity before 6 weeks after childbirth at *p*-value < 0.05 with 95% confidence interval.

This study revealed that participants with monogamy type of marriage were 4.4 times more likely to return to sexual activity before 6 weeks after childbirth than mothers whose type of marriage was polygamy [AOR = 4.4, 95% CI (2.1, 9.4)].

The study also showed that multiparous and grand-multi parous women were 89% and 90% less likely to have early postpartum intercourse [AOR= 0.11, 95% CI = (0.02, 0.81) and AOR = 0.10, 95% CI = (0.015, 0.72)].

Correspondingly, women who had postnatal care visit for the last births were about 2 times more likely to resume sexual intercourse within 6 weeks of childbirth than mothers those who had no postnatal care visit for the last births (AOR = 1.8, 95% CI (1.01–3) and postpartum women using formula feeding as infant feeding were 2.3 times more likely to resume sexual intercourse within 6 weeks of childbirth than mothers with exclusive breastfeeding (AOR = 2.3, 95% CI (1.3–4). (*See* Table 5).

**Table 5:** Factors associated with return to sexual activity within 6 weeks of childbirth among married post-partum women attending Salale University specialized and teaching hospital, from September to October 2021 (*n* = 421).

**Variables**		**Return to sexual activity after childbirth**		**COR 95%CI**	**AOR 95%CI**
		**<6 weeks**	**>6 weeks**		
Type of Marriage	polygamy	20 (4.8%)	109 (25.9%)	1	1
	Monogamy	113 (26.8%)	179 (42.5%)	0.3 (0.17, 0.5)	4.4 (2.1, 9.4) ^*^
Duration of living together with husband	1–2 years	74 (17.6%)	139 (33.0%)	1	1
	3–5 years	35 (8.3%)	77 (18.3%)	0.63 (0.36, 1.1)	1.3 (0.64, 2.5)
	>5 years	24 (5.7%)	72 (17.1%)	0.73 (0.4, 1.4)	1.5 (0.7, 3.2)
Occupation	Government employee	41 (9.7%)	89 (21.1%)	1	1
	Self-employee	30 (7.1%)	59 (14%)	0.8 (.47, 1.4)	0.28 (0.9, 1.9)
	Housewife	52 (12.4%)	126 (29.9%)	0.9 (0.55, 1.5)	0.4 (0.12, 1.3)
	Others^**^	10 (2.4%)	14 (3.3%)	0.5 (0.2, 1.3)	0.3 (0.01, 1.1)
Number of pregnancies	Primi-gravid	120 (28.5%)	246 (58.4%)	1	1
	Multi gravid	13 (3.1%)	42 (10.0%)	0.64 (0.33, 1.23)	0.56 (0.2, 1.5)
Parity	Primi-Para	46 (11%)	102 (24.3%)		1
	Multi-Para	82 (19.5%)	182 (43.3%)	3.7 (0.85, 16)	0.11 (0.02, 0.81) ^*^
	Grand- Multi-Para	5 (1.2%)	3 (0.7%)	3.7 (0.86, 15.85)	0.1 (0.015, 0.72) ^*^
Index of pregnancy status	Planned	129 (30.6%)	270 (64.1%)	1	1
	Un planned	4 (1.0%)	18 (4.3%)	0.5 (0.15, 1.4)	3.8 (0.97, 14.9)
Mode of delivery	SVD without episiotomy	107 (25.4%)	211 (50.1%)		1
	SVD with episiotomy	16 (3.8%)	45 (10.7%)	0.6 (0.3, 1.3)	1.6 (0.6, 4)
	Cesarean Section	10 (2.4%)	32 (7.6%)	0.9 (0.35, 2.2)	0.9 (0.28, 2.7)
Postnatal care visit for the last births	Yes	93 (22.1%)	164 (39.0%)	1	1
	No	40 (9.5%)	124 (29.5%)	0.6 (0.4, 0.9)	1.8 (1.01, 3) ^*^
Currently infant feeding practice	Exclusive breast feeding	98 (23.3%)	181 (43.0%)	1	1
	Formula Feeding	35 (8.3%)	107 (25.4%)	0.6 (0.4, 0.95)	2.3 (1.3, 4) ^*^
Last sex trimester of pregnancy	First trimester	11 (3.1%)	10 (2.8%)	1	1
	Second trimester	41 (11.5%)	73 (20.6%)	0.38 (0.15, 094)	2.5 (0.9, 7)
	Third trimester	65 (18.3%)	155 (43.7%)	0.75 (0.46, 1.2)	1.1 (0.6, 1.8)
Help from health care providers	Yes	38 (9.0%)	67 (15.9%)	1	1
	No	95 (22.6%)	221 (52.5%)	0.76 (0.5, 1.2)	0.6 (0.27, 1.5)
Postpartum health education care provider	Get	33 (7.8%)	51 (12.1%)	1	1
	Not get	100 (23.8%)	237 (56.7%)	0.65 (0.4, 1.1)	2 (0.8, 5.1)

* *= Statistically significant at p <0.05; 1 = Reference;*

Others^**^ = student, Non-governmental organization (NGO) employee and daily laborer; COR, Crude Odds Ratio; AOR, Adjusted Odds Ratio and 95% CI, 95% Confidence Interval.

## Discussion

In this study, the prevalence of return of sexual activity within 6 weeks of childbirth was 31.6%. The study found that polygamy, number of deliveries (parity), postnatal care visits, and current infant feeding patterns were significantly associated with return of postpartum sexual activity within 6 weeks of childbirth. The prevalence of this study was comparable with the study done in Gondar (26.9%), Nekemte (20.2%), Uganda (21.5%), and Nigeria (21.9%) (16–19).

The finding of this study was much lower than the findings of study conducted in Jimma town (53.9%), Uganda (66.4%), and Turkey (79.1%) (3, 12, 13). A possible reason for the difference may be due to differences in sociocultural differences. Another reason for the discrepancy could be where the study was conducted [specialized hospital versus (vs.) district hospital] and the differences in study design between the Turkish and the current study (cross-sectional vs. cohort study design).

The result of this study showed that women with monogamous type of marriage were 4.4 times more likely to resume sexual activity within 6 weeks of childbirth compared to women who were in polygamous type of marriage [AOR = 4.4, 95% CI = (2.1, 9.4)]. This is in line with research conducted in Ethiopia's Jimma district, which showed that women in monogamous form of marriage were more likely to resume sexual activity earlier than women in polygamous marriage (3). This might be justified that partners with a polygamous type of marriage have the option to have sexual intercourse with one of the wives, while postpartum mothers can abstain.

The study also showed that multiparous and grand-multi parous women were 89% and 90% less likely to have early postpartum intercourse [AOR = 0.11, 95% CI = (0.02, 0.81) and AOR =0.10, 95% CI = (0.015, 0.72)] compared with primiparous women, respectively. This is comparable to studies conducted at Aminu Kano Teaching Hospital (AKTH) in Nigeria, National Referral and Teaching Hospital in Murago Uganda, Nekemte town in western Ethiopia and Gondar city in northwestern Ethiopia (7, 16, 18, 19). These studies show that women with only one child return to sexual activity earlier than women with many children. The likely justification for this might be that women's wish to have another child within a short period of time.

Similarly, the current study also showed that women who had the postnatal care visit for the last births were about 2 times more likely to resume sexual intercourse earlier than women who had no postnatal care visit [AOR =1.8, 95% CI: (1.01, 3)]. This response is supported by the study done in Gondar city, Northwest Ethiopia (19) which indicated that women who obtained postnatal care had higher odds to initiate early sexual intercourse than who did not. This may be because postpartum visits allow women to receive more information and counseling about exclusive breastfeeding and postpartum contraceptives, thereby preventing unwanted pregnancies. As a result, they can start sexual activity earlier without worrying about an unwanted pregnancy.

Those who were currently formula-fed as an infant feeding were 2.3 times more likely to resume sexual activity than women who exclusively breastfed [AOR = 2.3, 95% CI: (1.3, 4)]. This is consistent with an analysis of study from 17 Asian and African countries (24), which showed that postpartum infant feeding status was significantly associated with early onset of postpartum intercourse. The reason for our study could be that postpartum women who are not breastfeeding are more likely to engage sexual activity because they are not subjected to the negative effects of breastfeeding, which include physical, hormonal, and psychological changes in the body. They are also devoid of the side effects of low estrogen caused by high prolactin hormone from nursing, which cause decreased vaginal lubrication and vaginal epithelial atrophy, making physical arousal difficult and intercourse unpleasant. Dyspareunia is a prevalent sexual dysfunction among breastfeeding mothers (5, 25). The additional reason may be the poor relationship of the women with their male partners (26).

## Conclusion

In the current study, about one-third of postpartum women returned to sexual activity within 6 weeks of childbirth. Monogamous marriage, parity, postpartum visits at last delivery, and current infant feeding status were found to be significantly associated with return of sexual activity within 6 weeks of childbirth. Strengthening postpartum counseling regarding the appropriate time to resume sexual activity is crucial. Regular postpartum visits following deliveries should also be encouraged by health care providers.

## Data Availability Statement

The raw data supporting the conclusions of this article will be made available by the authors, without undue reservation.

## Ethics Statement

The studies involving human participants were reviewed and approved by Salale University Ethical Review Committee. Written informed consent to participate in this study was provided by the participants' legal guardian/next of kin.

## Author Contributions

DE, TE, and BD: conceptualization and investigation. DE, MA, and TE: data curation, formal analysis, and methodology. DE: funding acquisition, project administration, resources, and writing—original draft. BD and MA: software. TE: validation. DE and TE: visualization. DE and BD: writing—review and editing. All of the authors contributed to the proposal development, questionnaire development, data collecting process, and analysis. The final version of the manuscript was read by all authors, who gave their approval for it to be considered for publication.

## Funding

The research was funded by Salale University, and the funders had no involvement in the study design, data collection, analysis, publication decision, or article writing.

## Conflict of Interest

The authors declare that the research was conducted in the absence of any commercial or financial relationships that could be construed as a potential conflict of interest.

## Publisher's Note

All claims expressed in this article are solely those of the authors and do not necessarily represent those of their affiliated organizations, or those of the publisher, the editors and the reviewers. Any product that may be evaluated in this article, or claim that may be made by its manufacturer, is not guaranteed or endorsed by the publisher.

## Abbreviations

AOR: adjusted odds ratio
CI: confidence interval
COR: crude odds ratio
IRB: institutional review board
PNC: postnatal care
SVD: spontaneous vaginal delivery
SD: standard deviation
WHO: world health organization
SPSS: statistical package for the social sciences.
